# Coinfections of the Respiratory Tract: Viral Competition for Resources

**DOI:** 10.1371/journal.pone.0155589

**Published:** 2016-05-19

**Authors:** Lubna Pinky, Hana M. Dobrovolny

**Affiliations:** Physics and Astronomy Department, Texas Christian University, Fort Worth, Texas, United States of America; University of Georgia, UNITED STATES

## Abstract

Studies have shown that simultaneous infection of the respiratory tract with at least two viruses is common in hospitalized patients, although it is not clear whether these infections are more or less severe than single virus infections. We use a mathematical model to study the dynamics of viral coinfection of the respiratory tract in an effort to understand the kinetics of these infections. Specifically, we use our model to investigate coinfections of influenza, respiratory syncytial virus, rhinovirus, parainfluenza virus, and human metapneumovirus. Our study shows that during coinfections, one virus can block another simply by being the first to infect the available host cells; there is no need for viral interference through immune response interactions. We use the model to calculate the duration of detectable coinfection and examine how it varies as initial viral dose and time of infection are varied. We find that rhinovirus, the fastest-growing virus, reduces replication of the remaining viruses during a coinfection, while parainfluenza virus, the slowest-growing virus is suppressed in the presence of other viruses.

## Introduction

Respiratory virus infections are a leading cause of mortality worldwide [1]. In addition to the threat from single infections, infections with multiple respiratory viruses in the same patient have been reported in many studies [2–11]. A number of respiratory viruses have been found to be capable of participating in simultaneous infections including respiratory syncytial virus (RSV), human rhinovirus (hRV), human enterovirus (hEV), influenza A virus (IAV), influenza B virus (IBV), human metapneumovirus (hMPV), coronavirus (CoV), parainfluenza virus (PIV), adenovirus (AdV), and human bocavirus (hBoV) [3, 5, 8]. It has long been known that simultaneous viral infections exhibit a phenomenon called viral interference where one virus blocks the growth of another virus [12–15], so the common observation of simultaneous respiratory infections in patients is somewhat surprising and needs explanation.

Children are the most common victims of simultaneous virus infections. An investigation by Goka et al. [3] with a study population ranging in age from 0 to 105 years reported that children aged less than 5 years show a higher propensity for viral coinfection than others. Another study found that the rate of viral coinfection is higher in children between 6–24 months [5] compared to new born babies (0–6 months). Finally, Zhang et al. [8] reported that among 164 children under 3 years of age, the 13–24 month age group had significantly higher multiple virus infections than the 8–12 month or 25–36 month age groups.

The severity of viral coinfections on clinical outcome in these patients is still unclear. Several investigations concluded that viral coinfections are no more severe than single virus infections [6, 7, 9], or even that there is less severe clinical impact associated with coinfection [5, 6]. On the contrary, some studies have evidence of severe disease outcome from viral coinfections [2, 3]. As an example of the confusion surrounding this issue, Aberle et al. [16] found that the severity of dual infections with non-RSV respiratory viruses are similar to those of single infections, whereas coinfection with RSV is associated with reduced immune responses resulting in a more severe clinical course of lower respiratory tract diseases. Brand et al. [7] also found that RSV associated coinfections are more severe than single RSV infections. Coinfections with influenza A and B viruses also appear to increase severity, leading to higher rates of admission to intensive care units or death [4].

To date, there are few experimental studies of simultaneous respiratory infections. One study examined co-infection of Reovirus and SARS coronavirus in guinea pigs, finding that a coinfection led to rapid death of the animals [17]. Another study examined coinfections of swine influenza and porcine reproductive and respiratory syndrome virus in vitro [18]. This study observed viral interference, but noted that the effect was dependent on which virus was the primary infection. There is only a single in vitro experiment that examines simultaneous infection of human respiratory tract viruses [19]. Shinjoh et al. showed that Influenza A virus has the potential to block the growth of RSV if they are likely to infect the host cells at the same time. In their experiment, RSV infection produces a higher peak viral load in single infection than in coinfection with influenza virus if the infections are initiated at the same time. Influenza multiplication can be suppressed by RSV, however, if the influenza infection is initiated after the RSV infection. They also analyzed this blocking action of one virus over another at the level of viral protein synthesis. During their experiments, immunofluorescense and scanning electron microscopy revealed that during the coinfection, both of the viruses release their specific surface antigens selectively indicating no viral interference involved in the blocking action.

In this paper, we use mathematical modeling to investigate simultaneous infections of the respiratory tract in an effort to explain these contradictory findings. We extend a simplified model of influenza infection [20] to include two viral strains and use it to gain insight into in vitro RSV and influenza coinfections. We then analyze other possible simultaneous viral infections in the respiratory tract with this model, focusing on more common pairs of simultaneous infections which include combinations of RSV, hRV, IAV, hMPV and PIV. We find that the period of coinfection for any of the combinations is no more than 10.5 d in the absence of any competitive advantage such as an increased initial viral inoculum or an earlier time of infection initiation.

## Results

### In vitro coinfection of RSV and IAV

**Fig 1:**
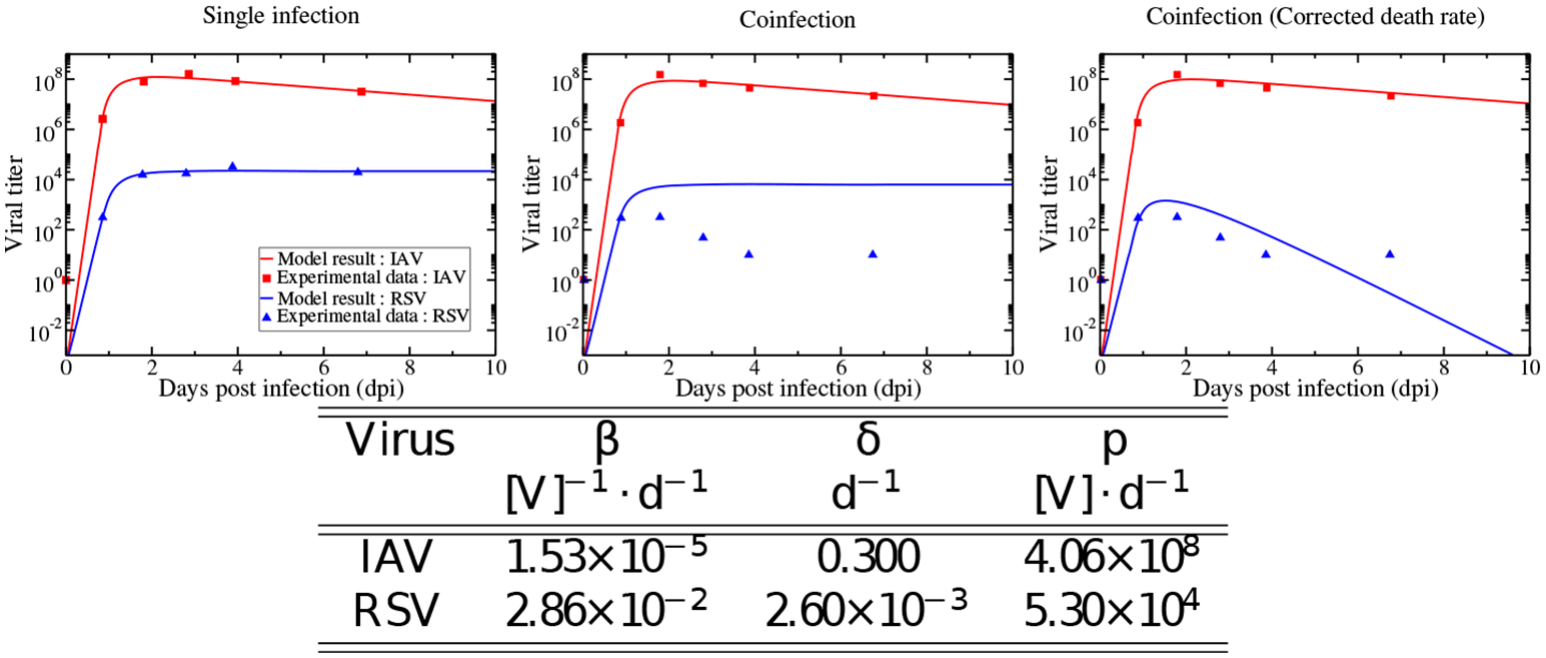
Model fits to the data from Shinjoh et al. [19]. (Left) Experimental data from single infections of RSV (blue) and influenza (red) are fit using a single infection model. Estimated parameters are given in the table (bottom). (Center) Coinfection model predictions and experimental data for RSV and influenza coinfection. (Right) Coinfection model with corrected decay rate predictions and experimental data for RSV and influenza coinfection.

While studies have shown that hospitalized patients commonly have evidence of infection by more than one virus at a single time [2–9], detailed studies of the time course of these infections has not yet been done. Our model will allow us to perform these detailed studies, but we must first ensure that it can reasonably reproduce experimental data of simultaneous infections. We use an in vitro experiment that studied coinfection of RSV and influenza A in MDCK cells [19] to test the whether our model can reproduce experimental observations. In the experiment, they initiated single infections of RSV and IAV individually in MDCK cells at an MOI of 0.001 and measured the viral titer in the supernatent over multiple time points (figure 1 in their paper).

We used the data (data available in S1 Table) from the single infection experiments of RSV and IAV to parameterize our model by fitting the reduced model of single virus infection. Since the data is limited, and not all parameters will be identifiable [21], we fix some of the parameters. We fix the initial number of target cells to 1 and fix the initial amount of virus to the MOI, since MOI is the initial ratio of infectious virus to target cells. Since the decay rate is determined by the smallest of *k*, *c*, or *δ* [22], only one of the three parameters is identifiable, so we fix *k* = 4.0 /d [20] and *c* = 2.4 /d [23] for both of them. While these are previously estimated values for influenza, we use the same values for RSV since there are no current estimates of these parameters for in vitro RSV infections and the values will not affect the outcome as long as *δ* is the smallest of the three quantities.

The resulting fits are shown in Fig 1 (left) and remaining estimated parameters are given in Fig 1 (bottom). RSV and influenza A virus reach their peaks at almost the same time post infection. The peak viral load for influenza is approximately 1 × 10^8^ PFU/mL and that for RSV is 1 × 10^4^ TCID_50_/mL. Influenza A virus produces greater viral load and has a faster initial growth rate than RSV in a single infection. Note that the experimental data for RSV does not show decay of the viral titer, so we cannot accurately estimate the true viral decay rate of RSV.

Now we use the proposed coinfection model to predict the kinetics of coinfection with RSV and IAV. We simulate the coinfection by starting the RSV and IAV infections at the same time with the same amount of initial viral inoculum, as was done in the experiment. The model predictions of the coinfection along with the experimental data are shown in Fig 1 (center). The model is able to correctly predict the influenza time course, but does not correctly capture the time course of the RSV infection. This is due to the poor estimate of viral decay rate of RSV. If we set *δ* = 2.0 /d, to more accurately reflect the actual decay rate of RSV, then the model shows a good agreement with the experimental data (Fig 1, right). Thus the model generates almost the same growth profile of coinfection as the experiment.

**Fig 2:**
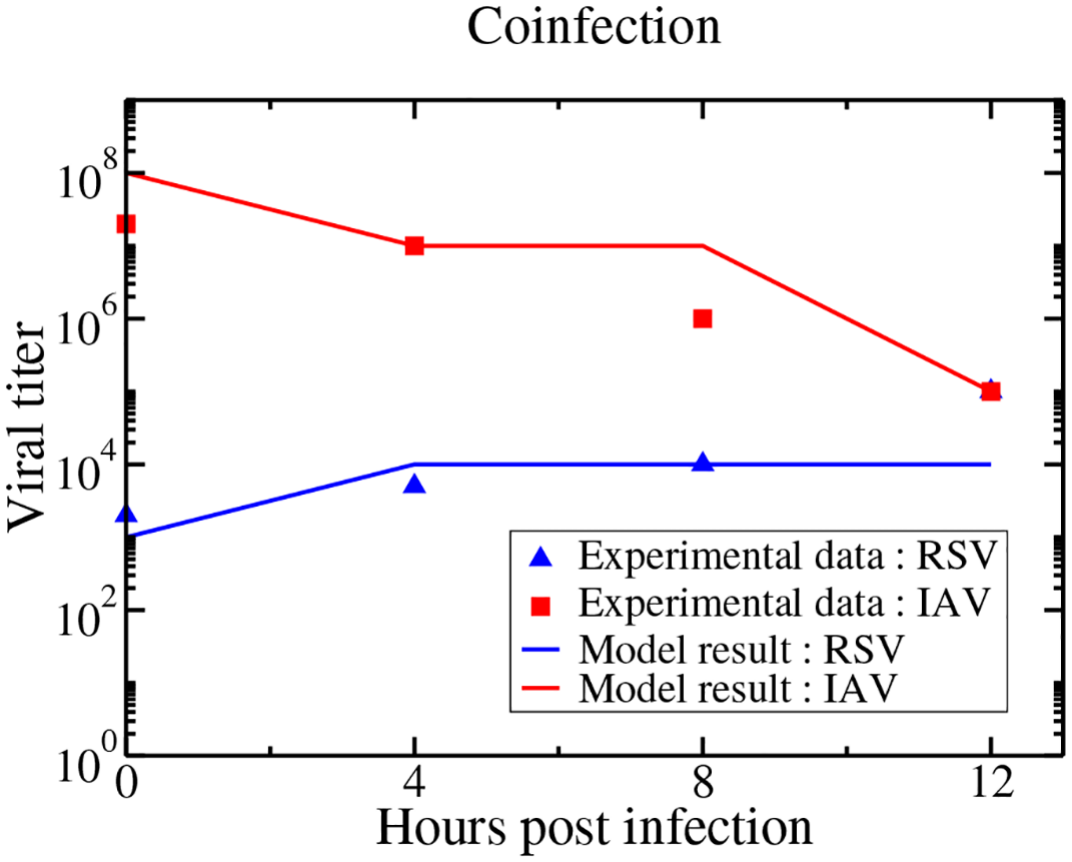
Delayed influenza infection. Our model predictions and experimental viral titer measurements of RSV and IAV viral titers measured at 51 hours post-RSV infection with IAV started with a delay of 0, 4, 8, and 12 h.

A second series of experiments was performed by Shinjoh et al. [19], in which they initiated an RSV infection in the MDCK cells at an MOI of 0.001 and then added influenza virus 0, 4, 8, or 12 h later at an MOI of 0.001 each time. They then measured the viral titers of both RSV and influenza in the supernatent at 51 h post-RSV infection. Our model predictions as well as the experimental data are shown in Fig 2. While our model predictions don’t exactly match the experimental measurements, we see the same trend of increasing RSV viral load and decreasing IAV viral load. Given the inherent error in viral titer measurements [24] and the difficulty in reproducing experimental results due to lack of unit standardization [25], our model manages to reproduce the data fairly well.

### Coinfections with other respiratory viruses

**Fig 3:**
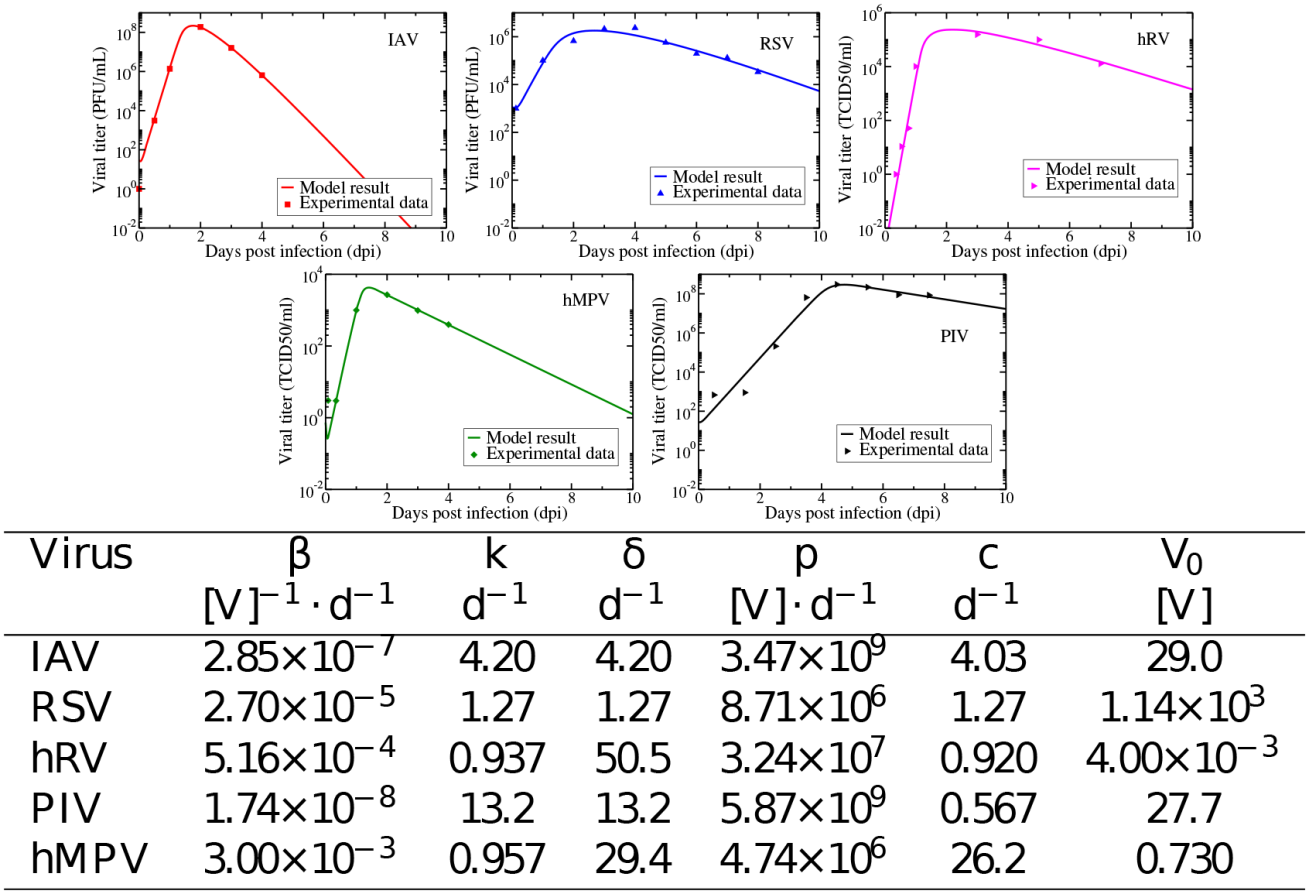
Single virus model fits to in vitro infections of respiratory tract cells. Experimental data and single virus model best fits for influenza (top left), RSV (top center), rhinovirus (top right), hMPV (bottom left) and parainfluenza (bottom right).

Now that we have seen that our model can predict the time course of a coinfection, we can use it with some confidence to predict the time courses of other combinations of respiratory viruses. We do not use the parameters estimated from the Shinjoh data in the remainder of the paper, but rather estimate new parameters for several different respiratory tract viruses. We first need to collect viral time courses for each of the viruses in a single infection. We require that the viral time course have both a growth phase and a decay phase, so that we can accurately estimate the decay rate of the virus, and that the infection takes place in human respiratory tract cells. Although a number of other viruses have been found to occur as part of simultaneous respiratory infections, we found suitable time courses for influenza A, RSV, rhinovirus, parainfluenza, and hMPV. We fit the single virus model to each of these data sets. For these fits, we fixed *T*_0_ = 1, but left the initial viral inoculum as a free parameter. We also did not fix any of *k*, *c*, or *δ* for any of the viruses although, as mentioned before, not all three are independently identifiable [22]. The resulting fits and estimated parameters are shown in Fig 3.

To our knowledge, viral kinetic parameters have not yet been estimated for in vitro RSV, hRV, PIV, or hMPV infections. Since viral units are not standardized, it is difficult to compare some of the parameters across the different viruses. Instead, we examine parameters such as viral growth rate, infecting time, viral decay rate and basic reproduction number to compare the kinetics of the viruses. Viral growth rate is calculated based on the equation derived by Smith et al. [22]. Smith et al. also determined that for this model, viral decay rate is given by the smallest of the decay parameters, *k*, *c* and *δ*. The infecting time, $t_{inf}=\sqrt{2/p\beta }$, represents the time it takes for a newly produced infectious particle to infect a susceptible cell [26]. The basic reproduction number represents the number of secondary infected cells that are produced from a single infectious cell and is given by *R*_0_ = *pβ*/*cδ* for this model [20]. The viral kinetics parameters for each respiratory virus are summerized in Table 1.

**Table 1:** Kinetic parameters of various respiratory infections.

Parameter	IAV	RSV	hRV	PIV	hMPV
Growth rate^a^ (/d)	11.9	5.41	13.6	3.99	9.07
Infecting time (d)	0.04	0.09	0.01	0.14	0.01
Decay rate (/d)	4.03	1.27	0.92	0.56	0.95
*R*_0_	58.4	146	360	13.8	18.4

^a^ Growth rate is calculated using the approximation derived by Smith et al.

Influenza kinetics parameters have been estimated before [23, 25, 27], and our parameter estimates lie within the range of previous estimates. Some of these parameters have also been estimated for RSV, although they are derived from in vivo patient data [28]. Our infecting time estimate of 0.09 d is similar to the 0.1 d estimate from Gonzalez-Parra et al. [28], but our estimated decay rate is smaller than the estimated in vivo decay rate. This is not unexpected since virus in patients is cleared faster due to the effect of the immune response. For the remaining viruses, these are, to our knowledge, the first estimates of viral kinetics parameters. Our estimates indicate that hRV is the fastest growing virus, followed by IAV and hMPV; RSV and PIV have much slower growth rates. RSV and PIV also have longer infecting times than the remaining viruses confirming that these two viruses spread more slowly through the cell population than the remaining three. Influenza has a much higher decay rate than the remaining viruses, while PIV has the smallest decay rate. The basic reproductive number of hRV is largest, indicating that it spreads easily within the respiratory tract, while PIV has the lowest reproductive number suggesting slower spread through the cell population.

**Fig 4:**
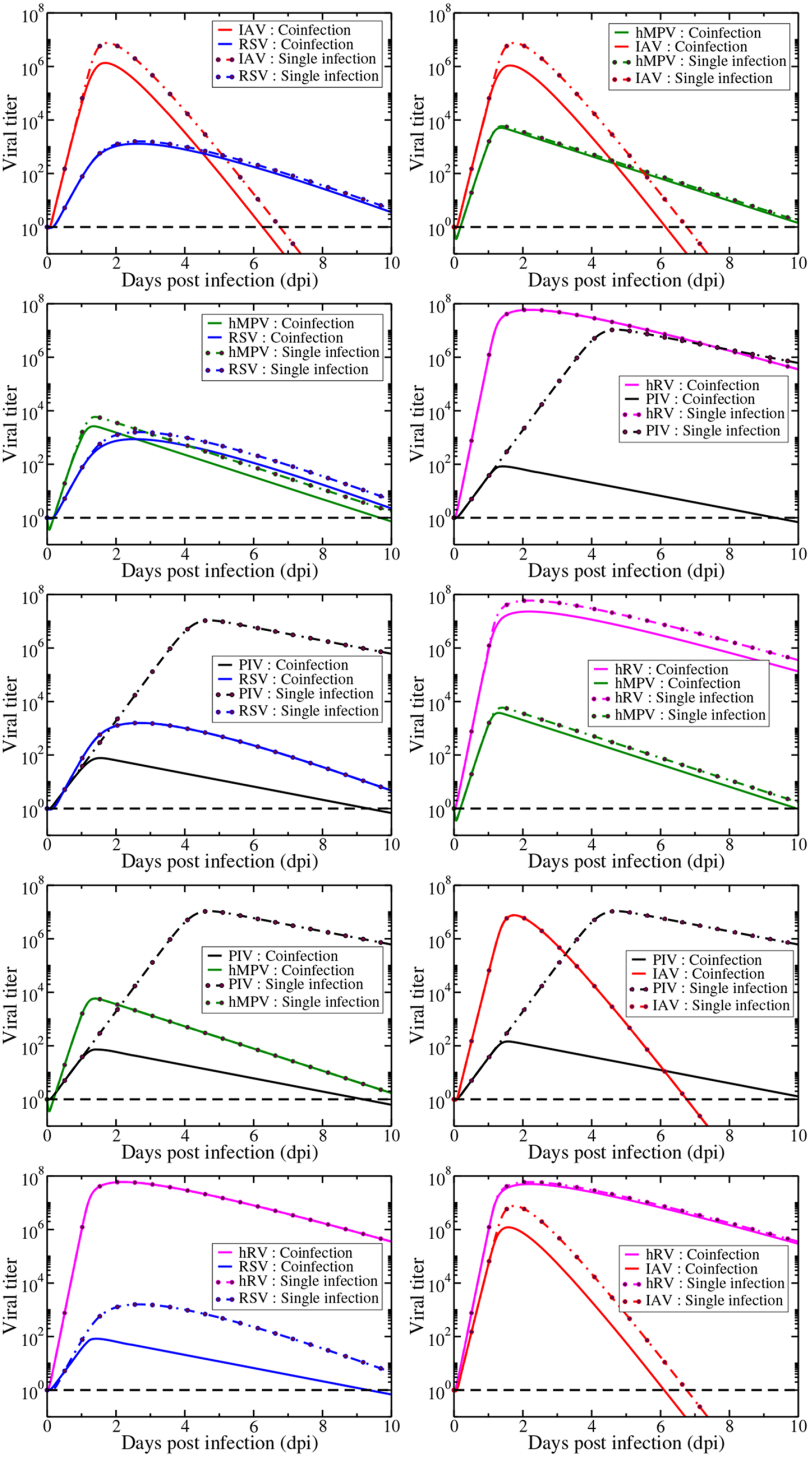
Model predictions of the time courses of simultaneous respiratory viral infections. Infections are initiated at the same time with the same amount of virus. Solid lines indicate the viral titer during a simultaneous infection while dashed lines indicate the viral titer during a single infection. The dashed black line indicates a typical experimental threshold of detection.

We can now use our two virus model to see how the viruses fare when they compete for target cells. In the simulations, infections with both viruses are initiated simultaneously with the same initial viral inoculum. Model predictions of time courses of all possible combinations of viral pairs in simultaneous infections are shown in Fig 4.

We see a wide variety of behaviors when respiratory viruses participate in coinfections. Rhinovirus growth (magenta curves) is largely unaffected by the presence of other viruses while replication of other viruses is diminished when rhinovirus is present. The initial growth rate for rhinovirus is the highest among all the viruses studied here, so rhinovirus will infect the available target cells more rapidly than the other viruses. hMPV, has a growth rate close to that of rhinovirus, and we see that it is the only virus that causes a visible decrease in the replication of rhinovirus (Fig 4 (third row, right)). At the other extreme, PIV (black curves) has the smallest growth rate of any of the viruses, and its replication is greatly inhibited by the presence of any other virus. These predictions indicate that the model suggests that simultaneous viral infections are a competition for the resource of target cells and that the virus with the largest growth rate will out-compete viruses with slower growth rates. In this way, growth of viruses with a slow growth rate can be blocked by a more rapidly growing virus. Unfortunately, growth of a virus with a fast growth rate will not be altered much by the presence of a slower growing virus. When viruses have comparable growth rates, the competition between the two will reduce replication of both viruses.

**Fig 5:**
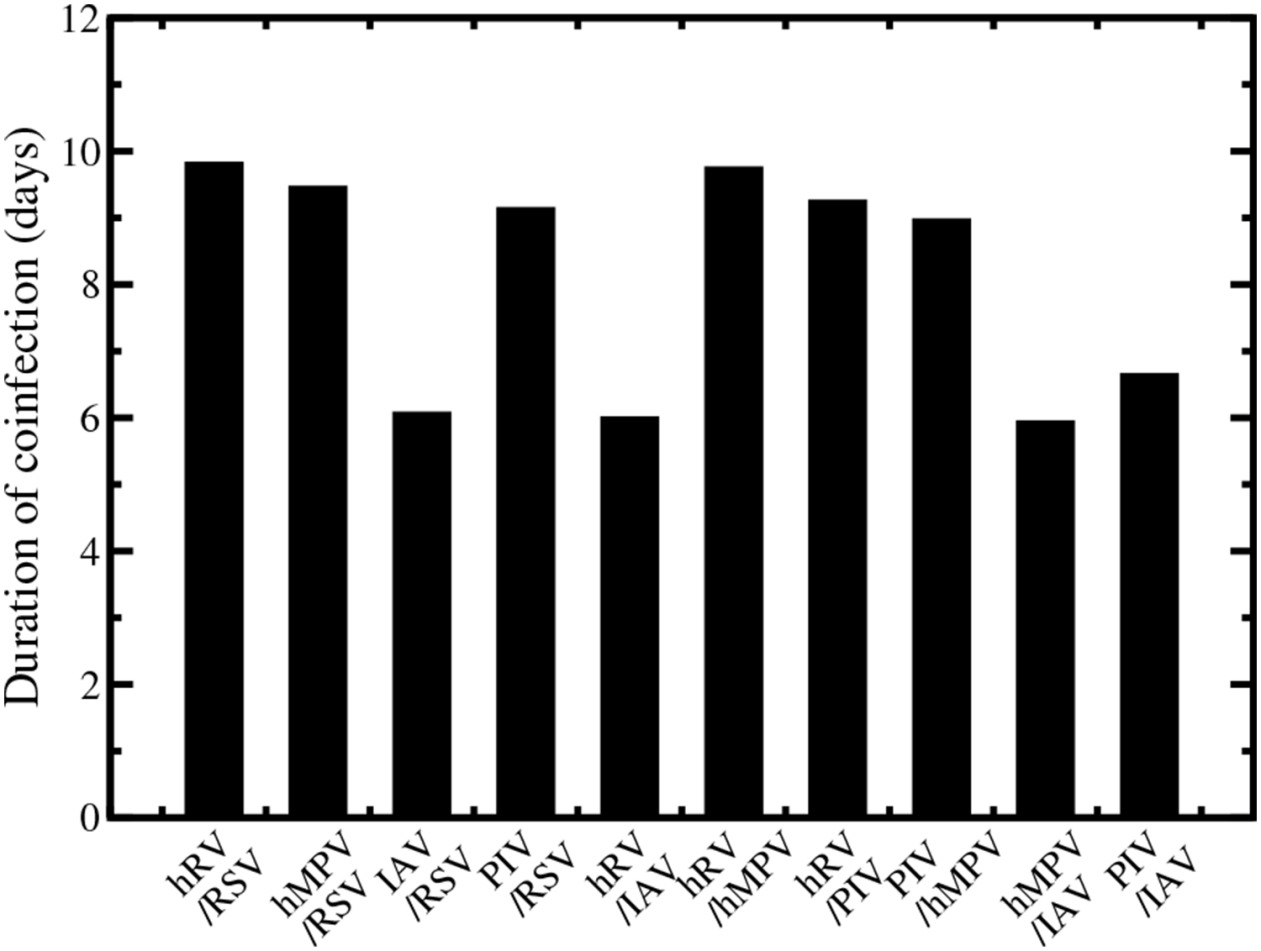
Duration of coinfection for each pair of viruses. Infections are initiated with the same amount of virus at the same time. Single infections are given by the dashed lines and coninfection dynamics are given by the solid lines.

Using our model, we can calculate the predicted duration of coinfection for each combination of simultaneous infections. We define coinfection here as the time during which both viruses have a viral load above the detection limit (dashed lines in the figures). The durations of coinfection for each pair of simultaneous infection are given in Fig 5. Our model predicts that even the longest simultaneous infections will be detectable for at most 10 days and that the shortest coinfections will be detectable for 6 days. This window of detectable coinfections explains why so many coinfections are being detected in patients [2–9].

#### Giving a competitive advantage

The scenario simulated in the previous section, where infections were started simultaneously with the same viral inoculum might be possible in an in vitro experiment, but in patients, this scenario is highly unlikely. A likely scenario for in vivo coinfections is that a patient starts to experience symptoms from the first infection and visits their doctor or the emergency room where they come into contact with the second virus. These infections are almost certainly not initiated with identical viral inocula either. However, these types of inequalities give a competitive advantage to one of the viruses. If one virus starts replicating before the other appears, it will have unfettered access to all the target cells until the second virus appears. Similarly, a larger initial viral inoculum allows one virus to infect more target cells in the first round of infection, leading to the production of more virus and so on. In this section, we examine the effect of different initial inocula and delayed initiation of the second infection. Since we simply need a single viral combination to use as a test case, we decided to use the viral combination found most often in patients, RSV and rhinovirus [3, 7]. Summary results for the remaining viral combinations are included in S1 Text.

**Fig 6:**
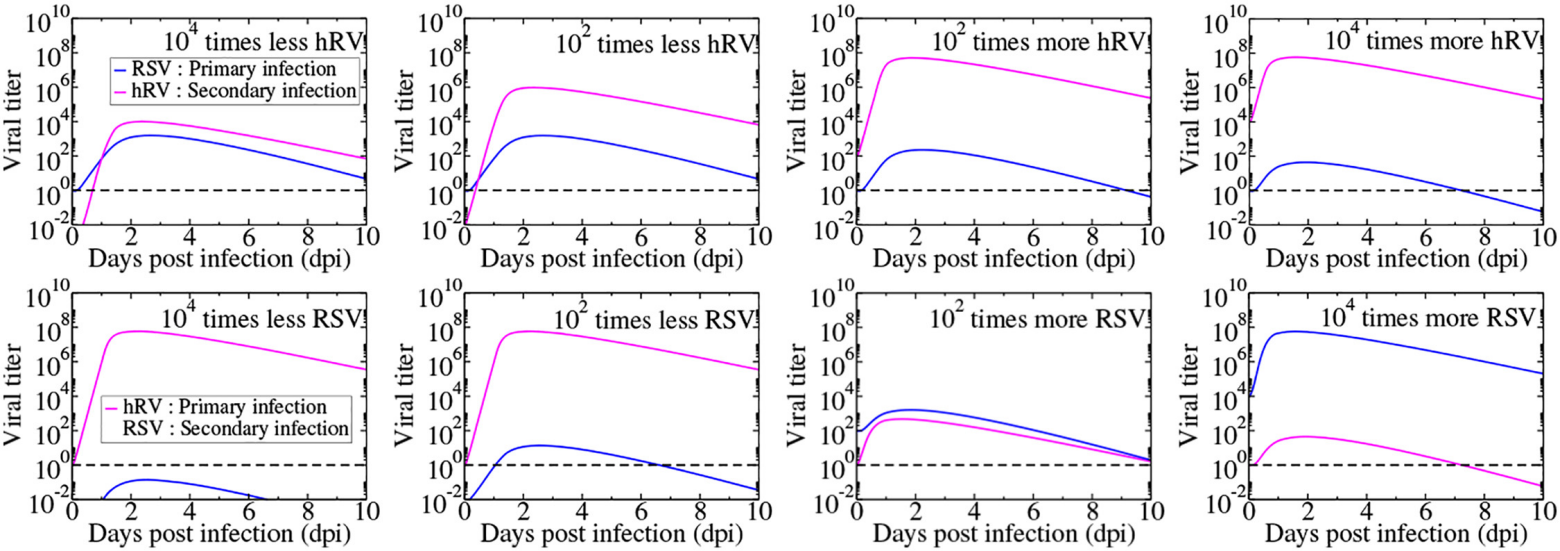
Simultaneous infection of rhinovirus and RSV when initial viral inoculum is varied. In the top row, the RSV inoculum is fixed and rHV inoculum is varied. In the bottom row, hRV inoculum is fixed and RSV inoculum is varied. The dashed line indicates a typical experimental threshold of detection.

The amount of virus used to initiate an infection plays an important role in deciding infection outcomes [29]. We varied the initial viral inoculum by fixing one viral inoculum and varying the other and then fixing the other viral inoculum and varying the first. Fig 6 shows the coinfection dynamics of RSV and rhinovirus when the initial viral inoculum is different. We see that if the RSV inoculum is large compared to the hRV inoculum, then RSV can suppress the growth of rhinovirus. While hRV can suppress growth of RSV for a wide range of initial inoculum conditions, it will only prevent it from growing past the detection threshold for very low inocula of RSV.

**Fig 7:**
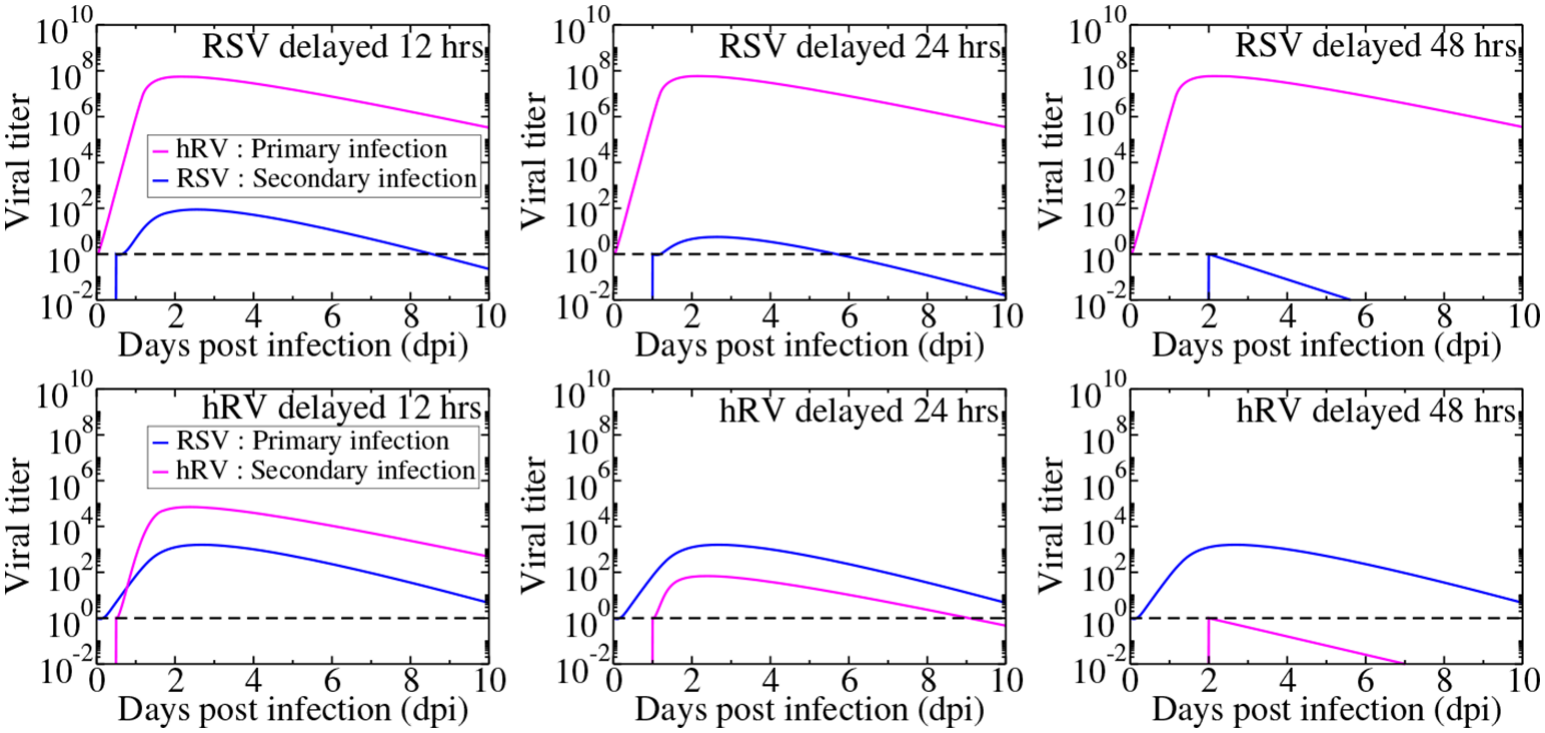
Simultaneous infection of rhinovirus and RSV with various time delays between the initiation of the infections. The dashed line indicates a typical experimental threshold of detection.

Another way to change the competitive advantage is to vary the starting times for the viruses. This is shown in Fig 7 for RSV and hRV infections. Not surprisingly, an initial rhinovirus infection can block RSV infection when the start of the RSV infection is delayed. An initial RSV infection can also block a rhinovirus infection if there is a sufficient time delay in the start of hRV infection. If the delay of hRV infection is long enough, RSV will have time to infect all of the target cells, leaving the rhinovirus with no resources to grow and the initial hRV inoculum will simply decay. More generally, if the start of the second infection is delayed too long, the first infection uses up all the target cells, suppressing the secondary infection.

**Fig 8:**
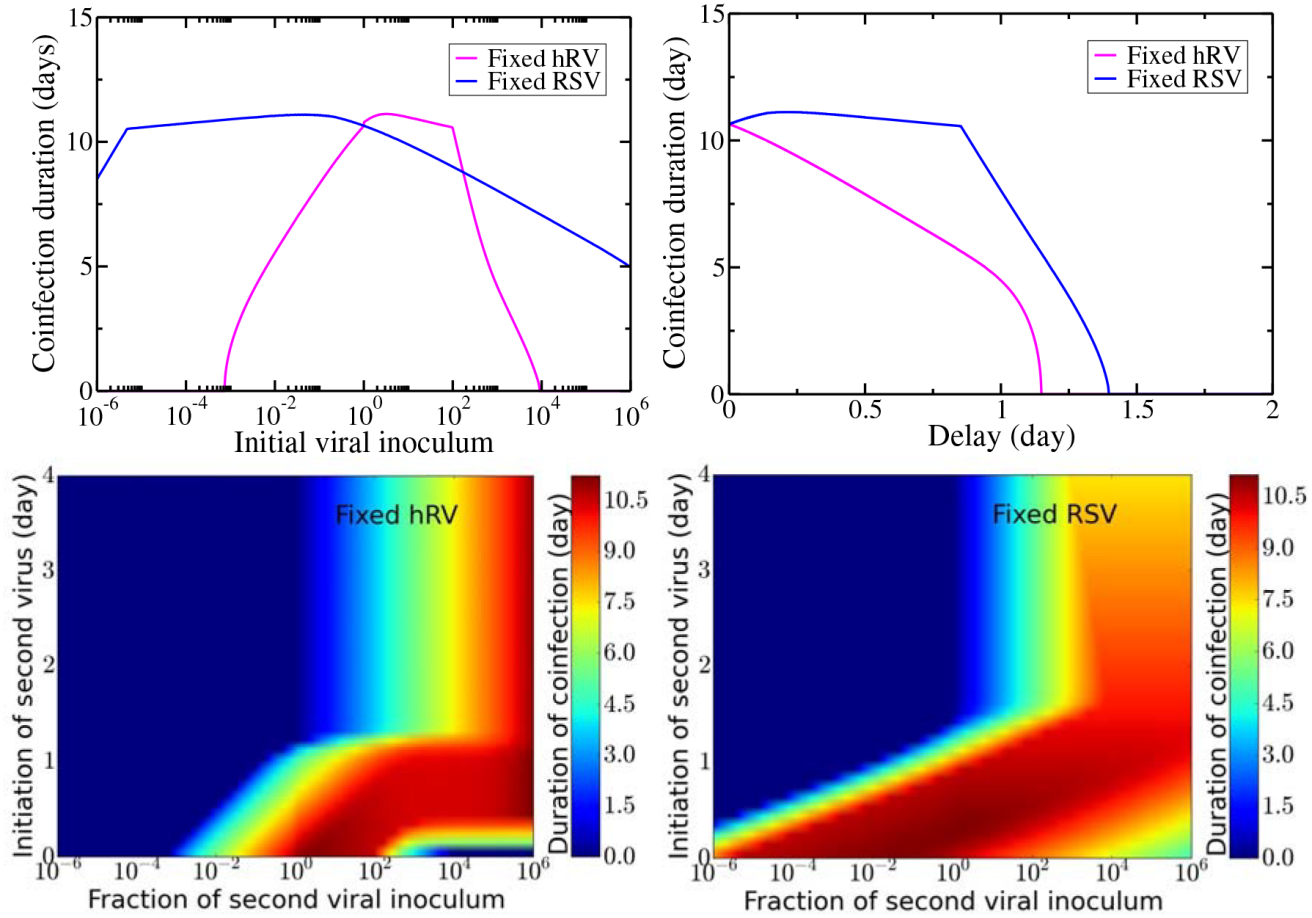
Coinfection duration with varying initial viral inoculum and relative starting time of infection. Coinfection duration as a function of initial viral inoculum (top left), relative starting time (top right) and as a function of both with hRV infection fixed (bottom left) and RSV infection fixed (bottom right).

Fig 8 illustrates the duration of coinfection for each of these competitive advantages. For fixed hRV inoculum, coinfection is only possible when the initial dose of RSV is approximately equal to the hRV inoculum, while for fixed RSV inoculum, coinfection is possible for almost all the combinations of viral inocula (Fig 8, top left). Fig 8 (top right) gives the coinfection duration as a function of delay if the initial viral inocula are identical. When hRV is the primary infection, the coinfection duration declines more quickly than when RSV is the primary infection. When RSV is the primary infection, the hRV needs a delay of ∼24 h to start reducing the duration of coinfection. In both cases, there is a maximum time delay beyond which coinfections will not occur since all target cells have been infected by the primary virus so the secondary virus cannot grow. Fig 8 (bottom) shows the coinfection duration as a function of both the ratio of initial inocula (*x* axis) and the delay in the start of the second infection (*y* axis) when hRV is the primary infection (bottom left) and when RSV is the primary infection (bottom right). Coinfection is possible only with certain combinations of initial viral dose and time delays with a maximum possible coinfection duration of ∼11 d. No matter which virus is the primary infection, we see that a large initial inoculum for the secondary virus will overcome even rather long time delays and will lead to long-lasting coinfections.

## Discussion

In this paper, we examined a mathematical model of simultaneous respiratory tract viral infections. We tested the model by reproducing the results of an in vitro experiment that examined an RSV and influenza A virus coinfection. Once an appropriate growth rate for RSV was used, the model correctly reproduced viral titers observed during a simultaneous RSV and IAV infection. The model also qualitatively reproduced the measurements of viral load when IAV infection was delayed. Once we had validated the model, we performed an independent study that predicted coinfection durations for various combinations of respiratory viruses. When predicting coinfection durations for combinations of IAV, RSV, hRV, hMPV, and PIV, parameters were estimated from experiments performed in respiratory tract cell cultures. Using these parameters, coinfection dynamics of different pairs of viruses were studied, providing predictions of coinfection dynamics in respiratory tract cells. In this model, the viruses compete for the resource of target cells, so the model predicts that viruses with a higher growth rate will out-compete viruses with a lower growth rate since the faster growing virus will consume more target cells early in the infection. This was seen by the unaltered growth of hRV, which has a high growth rate, in the presence of most of the other viruses. This was also seen in the suppression of replication of PIV, which has the lowest growth rate, when other viruses are present. We found that this competitive advantage could be overcome or amplified by allowing for different initial inocula or by delaying the start of one of the infections.

### Implications of our findings

Our key finding is that blocking of one virus infection by the presence of another can be explained simply through resource competition. Some studies have suggested that other mechanisms, such as the immune response [18, 19, 30, 31] or interference through viral proteins [31, 32] are responsible for the growth interference between two viruses. Our model does not include either of these mechanisms, suggesting that they are not necessary to explain the phenomenon. This does not mean, however, that these interactions do not play a role in coinfections, but that they should be considered in addition to resource competition. Interference through immune interactions can be considered a competitive advantage for one of the viruses in much the same way as we examined the effect of initial inoculum or delayed initiation of infection.

Our findings will have implications for the treatment of respiratory infections. Consider a simultaneous infection with influenza and RSV. There are treatments or drugs available for influenza infection [33] but not for RSV [34]. We found that influenza has a higher growth rate than RSV and so will hinder the replication of RSV, probably keeping RSV viral loads low, possibly below the detection level. A patient with this simultaneous infection goes to the doctor, who only detects the influenza infection and decides to treat the infection. Influenza replication is now suppressed by the drug, allowing the hidden RSV infection to emerge. RSV, of course, cannot be treated so the patient will end up suffering through the RSV infection. If the doctor had decided not to treat, influenza would have continued replicating, most likely suppressing the RSV infection.

We could also take advantage of the blocking action of fast-growing viruses. We clearly do not want to infect people with a virus that will make them sick, but there has already been some investigation into the use of defective interfering particles (DIPs) as agents to prevent or treat viral infections [35–37]. DIPs are viruses that contain genetic deletions rendering them unable to replicate on their own, but able to replicate in the presence of virus that provides the missing pieces of genetic material. When able to replicate, DIP-infected cells produce more DIPs than replication-competent virus. Our model suggests that as long as DIP growth is faster than that of the competent virus, it could block growth of the competent virus.

While we examined coinfections in the respiratory tract, this model can also be used to study coinfections in other systems. Multiple infections are not only prevalent in infections of respiratory tract, but also in the gastrointestinal tract [38, 39], liver [40, 41] and genital tract [42]. In nature, persistant viral infections have also been found with viruses such as phage viruses and badnavirus [43]. Our model’s prediction that viral growth rate determines which virus will dominate a simultaneous infection, will likely help to explain the dynamics seen in coinfections of these systems as the fundamental principle of resource competition is at play in these systems as well.

### Limitations of the model

As noted in the methods section, this is a rather simple model that makes some assumptions that simplify the biological complexity of the real system. For example, the contribution of the immune response to the interaction of the two viruses is not included. While there have been some attempts to incorporate the immune response into mathematical models of infection, for acute infections, experimental immune data is often too sparse to build accurate models [44]. We have not included the immune response in our model since there is little quantitative information about the immune response to the five viruses studied here, making it difficult to estimate the values of the extra parameters that would be necessary. Our model shows that viruses fundamentally interact through a competition for resources, but stimulating the immune response can potentially enhance or hinder the competitive advantage of one virus, with some studies suggesting that the immune response plays a role in viral interactions during coinfections [18, 19, 30, 31].

Cell regeneration was also not included in the model, but could potentially alter the dynamics of coinfection. In relatively short coinfections of the respiratory tract, cell regeneration largely does not take place until after clearance of the infection [45], but in longer-lasting infections such as hepatitis B and C coinfections [32], regeneration provides a steady supply of fresh target cells, limiting the competitive advantage of the fast-spreading virus. The addition of cell regeneration might make it possible for both viruses to co-exist for a long time leading to chronic coinfection.

We also assumed that there was no superinfection, or that two viruses cannot infect the same host cell simultaneously. While some experiments have observed superinfection exclusion [46] with the same strain, other experiments suggest that superinfection with different viruses is possible [19, 32]. If both viruses are able to coinfect cells, and more importantly, have the cell produce both types of virus, as observed by Shinjoh et al. [19] for RSV and IAV, then this eases some of the resource competition and might alter coinfection dynamics.

Conversely, our model assumes that both viruses infect the same type of cell. However, it is known that respiratory viruses do not necessarily infect the same respiratory cell type. Viruses are well known to exhibit tropism, which is determined by the nature of specific cell surface receptor activity of a virus during the binding process [47]. Two human influenza strains of H1N1 and H3N2 bind more strongly to tracheal and bronchial tissue whereas avian strains of H5N1 and H6N1 attach to type II pneumocytes and alveolar macrophages in the lower respiratory tract [48]. RSV has an affinity to bind with cell-surface nucleolin expression which has been reported to be found in different cell types including not only in respiratory tract but also tissues outside of the respiratory tract [49]. Another study found that hMPV infects primarily the ciliated respiratory epithelial cells [50]. It has also been reported that different types of parainfluenza viruses such as PIV1 and PIV3 are characterized according to their cell binding sites [51]. Rhinovirus were divided into two different groups (minor and major groups) of viruses who use different receptors for cell attachment [52]. In this respect, our investigation is limited by having all the participating viruses infect the same type of cell in the respiratory tract during coinfection given that cell surface receptor specificity for these viruses allows for variation in targeted cell populations. Clearly, if one of the viruses participating in the coinfection has access to target cells not accessible to the second virus it will have a competitive advantage. If the two target cell populations don’t overlap at all, then there will not be direct competition for resources, and the dynamics of the two viruses will be driven by their individual virus-cell interactions.

### Guidance for further experimental studies

Since our model neglected a number of factors that might play a role in the duration of coinfections, it would be helpful to have more experimental data to validate the model. While we did fit the model to the limited data available in the literature, more thorough testing of the model is needed. We have provided here predictions of coinfection durations for various combinations of five different respiratory viruses, any of which could be tested through experiments. The ideal experiment would consist of measuring viral titer and dead cells over time, sampling at a minimum of every 12 h, capturing several points during both the growth and decay phases of a single virus in vitro infection for two different viruses. We need both viral load and dead cells to properly identify model parameters for the two viruses [21]. We would then perform several in vitro coinfections with the same two viruses, varying the initial inocula of both viruses or introducing a time delay between the start of the two infections. For the coinfection, we would simply require measurement of the time course of viral titer of both viruses, again sampling frequently and over the entire duration of the infection. As we did here with the Shinjoh data, we would fit the single infection model to the single infection data and use the parameters to predict the dynamics of the resulting coinfections. The model predictions could then be compared to more extensive experimental coinfection data, either giving greater confidence in the model’s validity, or if the model fails to correctly predict viral time courses, motivating extension of the model to include more complexity.

The work presented here represents a first step in modeling respiratory virus coinfections. Our model predictions help elucidate the fundamental competition for resources that drives dynamics of respiratory coinfections, but there are many other factors that can change the competitive balance between the two viruses.

## Methods and Model

### Mathematical Model

We propose a model based on ordinary differential equations used for explaining influenza viral kinetics [20]. Our model represents the dynamics of simultaneous infection in the human respiratory tract with two viruses, *V*_1_ and *V*_2_. The model equations are

$$\begin{array}{cccccc} \text{Target}\;\text{cells}: & & \frac{dT}{dt} & =-\beta_{1}TV_{1}-\beta_{2}TV_{2}\\ \text{Eclipse}\;\text{cells}: & & \frac{dE_{1}}{dt} & =\beta_{1}TV_{1}-k_{1}E_{1} & \frac{dE_{2}}{dt} & =\beta_{2}TV_{2}-k_{2}E_{2}\\ \text{Infected}\;\text{cells}: & & \frac{dI_{1}}{dt} & =k_{1}E_{1}-\delta_{1}I_{1} & \frac{dI_{2}}{dt} & =k_{2}E_{2}-\delta_{2}I_{2}\\ \text{Virus}: & & \frac{dV_{1}}{dt} & =p_{1}I_{1}-c_{1}V_{1} & \frac{dV_{2}}{dt} & =p_{2}I_{2}-c_{2}V_{2}. \end{array}$$

**Fig 9:**
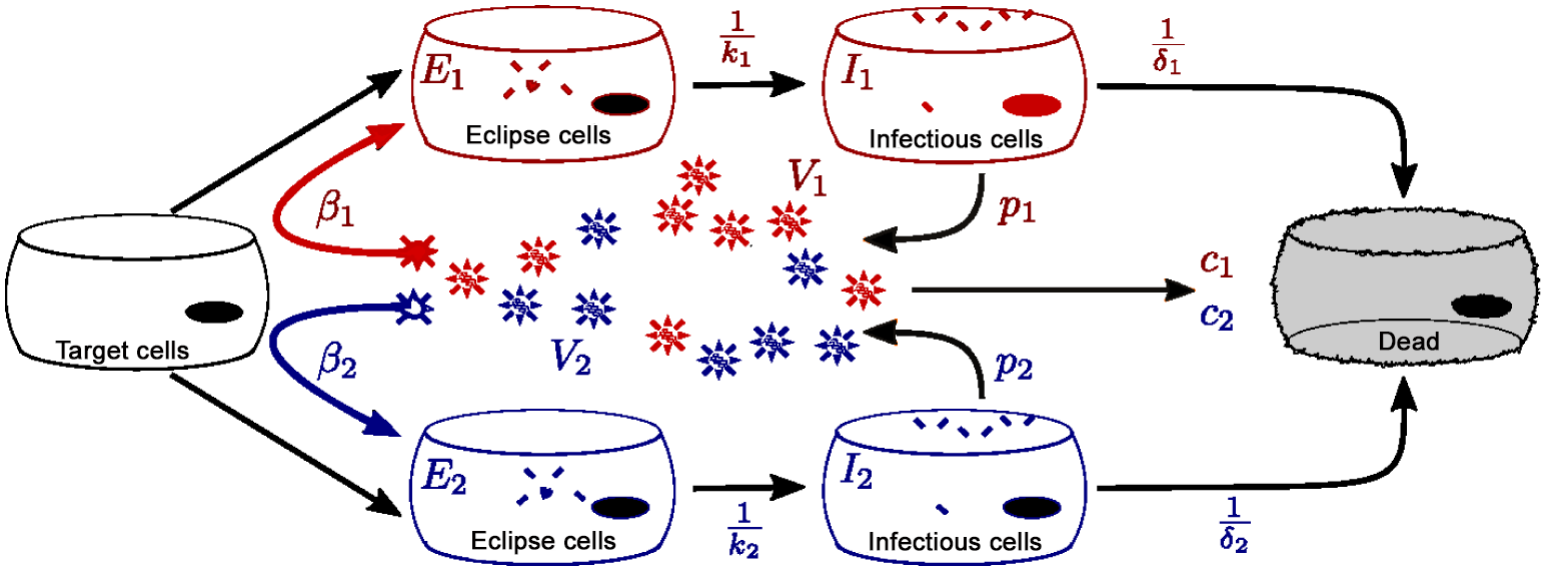
Mathematical model of simultaneous infection by two viruses. The two viruses infect the same target cell population, but coinfection of single cells is not allowed. Once infected, they enter an eclipse phase where they take some time before actively producing viruses. Newly produced viruses go on to infect other target cells.

Model parameters and variables are described in Table 2. As shown in Fig 9, *V*_1_ and *V*_2_ infect the susceptible uninfected target cells, *T*, at rates *β*_1_ and *β*_2_. We assume that one cell can only be infected by one type of virus at a time, i.e. *V*_1_ and *V*_2_ cannot simultaneously infect the same cell. The newly infected cells enter an eclipse phase, *E*_1_ or *E*_2_, where infected cells take some time to produce viral components. This delay accounts for intracellular processes related to the synthesis of viral nucleic acid and proteins, viral assembly, maturation and budding. After an average transition time $\frac{1}{k_{1}}$ or $\frac{1}{k_{2}}$, the cells become productively infectious cells, *I*_1_ and *I*_2_, which produce viruses at rates *p*_1_ and *p*_2_. The lengths of time over which infectious cells produce viruses are denoted by $\frac{1}{\delta_{1}}$ and $\frac{1}{\delta_{2}}$ after which the infectious cells die. Virus is cleared at rates *c*_1_ or *c*_2_. We assume both viruses attack the same type of target cell population, which is not always the true as respiratory virus infections also depend on the expressions of cell surface receptors [47, 49, 50]. Also, target cell regeneration is neglected here because infections are short compared to the time it takes for cells to regenerate [45]. No explicit immune response is considered in this model since accurate information about its role in viral infections is still lacking [44]. Finally, this model assumes exponential distributions for eclipse and infectious transition times, which is known to be biologically unrealistic [26, 53], but simplifies the computation and should not affect the qualitative predictions of the model. Our model is similar to those of [27, 54] except that they include non-exponential distributions as well as non infectious virus particles in their models.

**Table 2:** Parameter definitions of the model.

Parameter	Definition
T	number of uninfected target cells
E	population which is infected but not yet producing virus
I	population which is actively producing virus
V	infectious viral titer
*β*	infection rate
$\frac{1}{k}$	transition time from E to I
$\frac{1}{\delta }$	lifespan of infectious cells
*p*	rate of increase of viral titer per infectious cell
*c*	clearance rate of virus
*V*_0_	best fit initial virus titer
*T*_0_	amount of initial target cells

### Experimental data

We require experimental data for two purposes. We would like to test the ability of the model to reproduce experimental data from coinfections and we would then like to use the model to make predictions about coinfections with other respiratory viruses. To achieve the first goal, we looked for in vitro experiments studying simultaneous infections of respiratory viruses. The only experimental data we found was the RSV and influenza coinfection experiment by Shinjoh et al. [19]. In this experiment, they infect MDCK cells with a long strain of RSV and A/WSN/33 (H1N1) strain of influenza virus at a multiplicity of infection (MOI) of 0.001 both as single infections and as a simultaneous infection. While the quantitative data from this experiment is limited, we nonetheless try to use it as a minimal test of the validity of the model. The data extracted from the in vitro experiments of Shinjoh et. al. are available in S1 Table.

To achieve the second goal of this paper, we required experimental data of single respiratory virus infections. Our model has 5 parameters and requires that we have information on both the viral growth phase and the viral decay phase. In order to properly parameterize the model, we searched for viral titer measurements from in vitro multiple cycle infections performed in human respiratory tract cell lines which contain both growth and decay phases. These requirements for the data limited the number of viruses we could include in our study since we found suitable data only for IAV, RSV, hRV, PIV, and hMPV. When validating the model, the data used is taken from infections in MDCK cells while predicting viral time courses for combinations of IAV, RSV, hRV, hMPV, and PIV, parameters are to be estimated from experiments performed in respiratory tract cell cultures. A summary of the data sets used to parameterize respiratory viruses are shown in Table 3.

**Table 3:** Experimental data used to parameterize common respiratory tract infections.

Paper	Virus	Cell type
Danzy et al. [55]	Influenza NL/09	Human tracheo-bronchial epithelial
Liesman et al. [56]	RSV A2	Human airway epithelium
Yamamya et al. [57]	Rhinovirus 14	Human tracheal submucosal gland cells
Bartlett et al. [58]	Parainfluenza 1	Human airway epithelium
Scagnolari et al. [59]	hMPV NL-001	Human epithelial type 2

### Fitting procedure

Data were extracted using www.WebPlotDigitizer.com. We fit each data set with a single virus model using custom-written software in Octave 3.6.4 [60] that uses either the leasqr function, which uses Levenberg-Marquardt nonlinear regression, or the nelder_mead_min function which uses Nelder-Mead minimization to minimize SSR.

Confidence intervals for parameter fits are found through parametric bootstrapping [61]. 1000 surrogate data sets are generated by adding randomized errors to the best fit model prediction. The best fit to these new data sets are found using the same procedures described earlier. Resulting parameter values are ranked and used to determine the 95% confidence intervals.

## Supporting Information

S1 Text Coinfection duration as a function of initial viral inoculum (top left), delay (top right) and as a function of both for the remaining combinations of IAV, RSV, hRV, hMPV, and PIV.
(PDF)

S1 Table Shinjoh in vitro experimental viral load data.
(PDF)
