# Supplementary material for: Coinfections of the Respiratory Tract: Viral Competition for Resources
Source: PLoS One. 2016 May 19;11(5):e0155589. doi: 10.1371/journal.pone.0155589 (PMC4873262; doi:10.1371/journal.pone.0155589)
Supplement: S1 Text — Coinfection duration as a function of initial viral inoculum (top left), delay (top right) and as a function of both for the remaining combinations of IAV, RSV, hRV, hMPV, and PIV. (PDF) [file pone.0155589.s001.pdf]

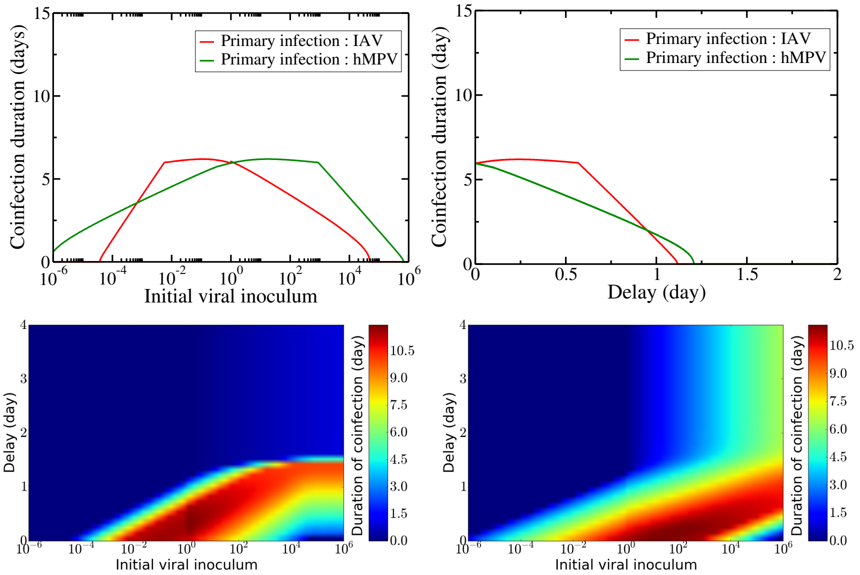

Figure 1: Coinfection duration as a function of initial viral inoculum (top left), delay (top right) and as a function of both for IAV as primary infection (bottom left) and hMPV as primary infection (bottom right).

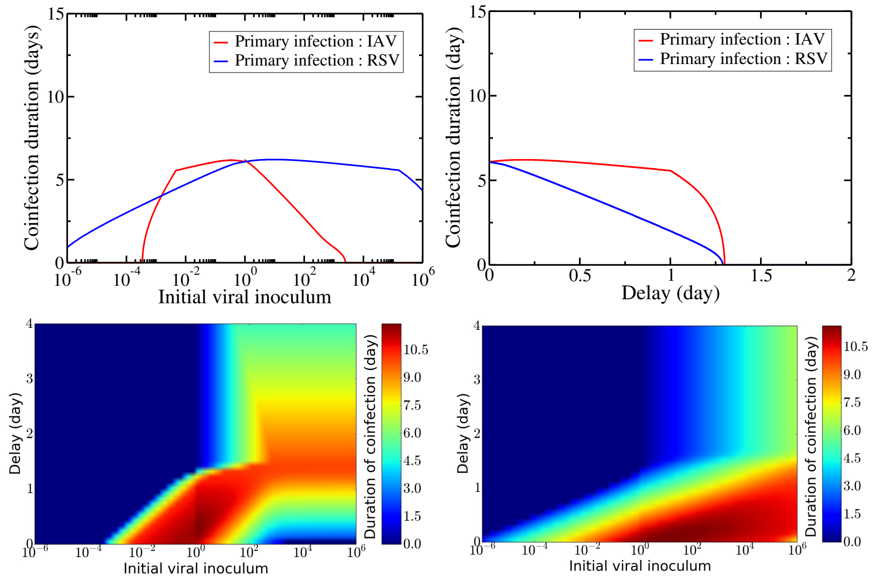

Figure 2: Coinfection duration as a function of initial viral inoculum (top left), delay (top right) and as a function of both for IAV as primary infection (bottom left) and RSV as primary infection (bottom right).

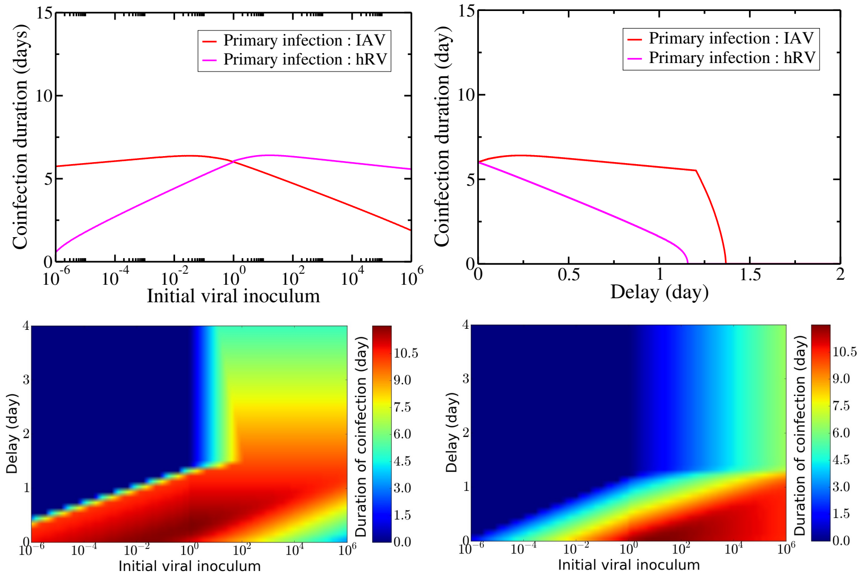

Figure 3: Coinfection duration as a function of initial viral inoculum (top left), delay (top right) and as a function of both for IAV as primary infection (bottom left) and hRV as primary infection (bottom right).

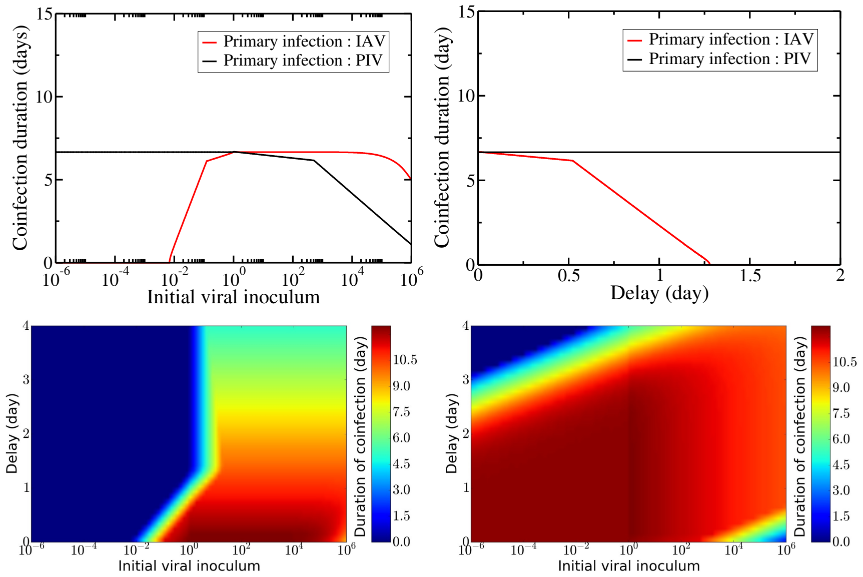

Figure 4: Coinfection duration as a function of initial viral inoculum (top left), delay (top right) and as a function of both for IAV as primary infection (bottom left) and PIV as primary infection (bottom right).

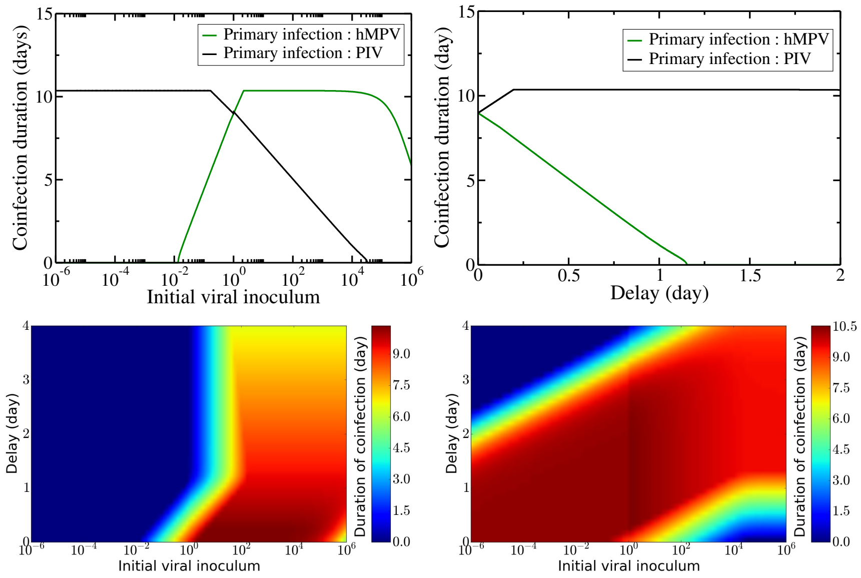

Figure 5: Coinfection duration as a function of initial viral inoculum (top left), delay (top right) and as a function of both for hMPV as primary infection (bottom left) and PIV as primary infection (bottom right).

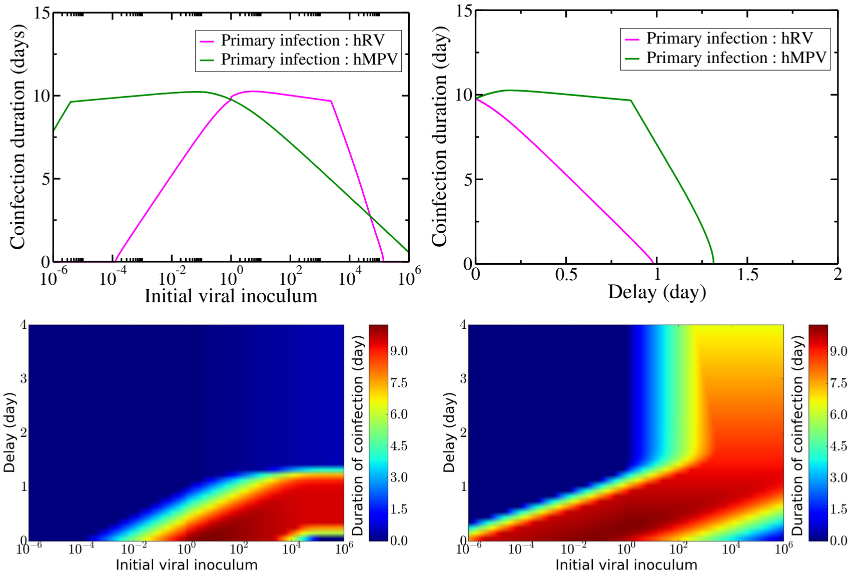

Figure 6: Coinfection duration as a function of initial viral inoculum (top left), delay (top right) and as a function of both for hRV as primary infection (bottom left) and hMPV as primary infection (bottom right).

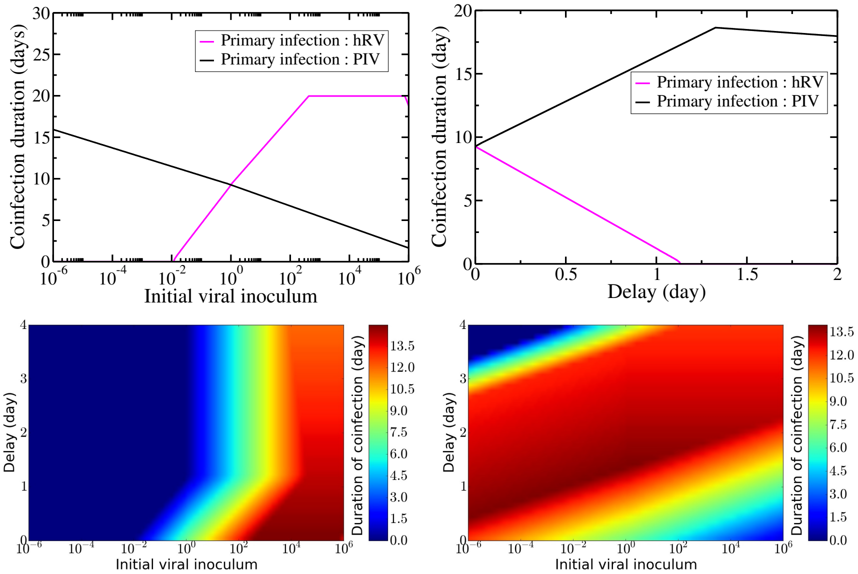

Figure 7: Coinfection duration as a function of initial viral inoculum (top left), delay (top right) and as a function of both for hRV as primary infection (bottom left) and PIV as primary infection (bottom right).

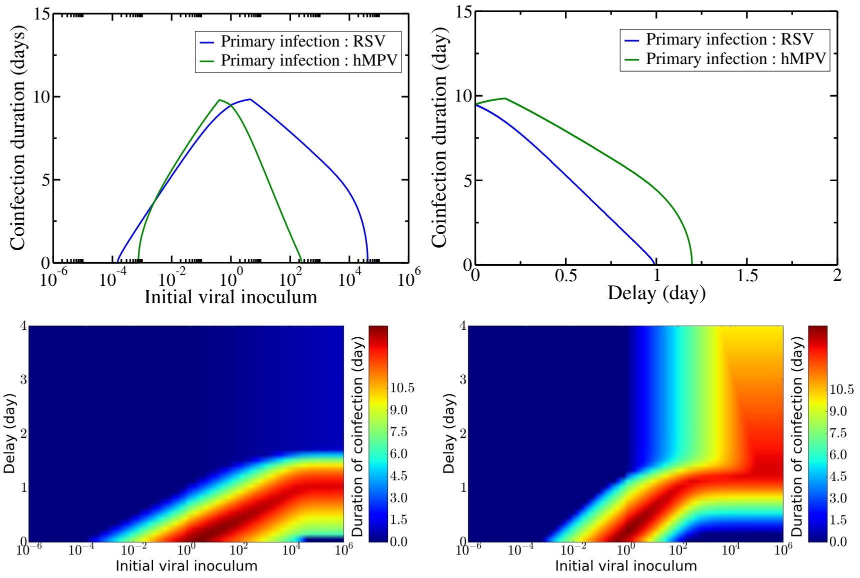

Figure 8: Coinfection duration as a function of initial viral inoculum (top left), delay (top right) and as a function of both for RSV as primary infection (bottom left) and hMPV as primary infection (bottom right).

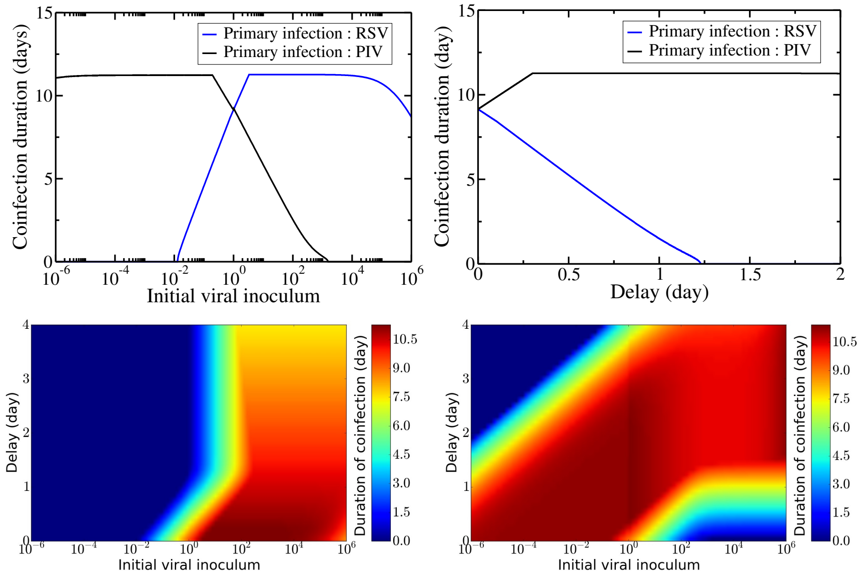

Figure 9: Coinfection duration as a function of initial viral inoculum (top left), delay (top right) and as a function of both for RSV as primary infection (bottom left) and PIV as primary infection (bottom right).
