# Supplementary material for: Coinfections of the Respiratory Tract: Viral Competition for Resources
Source: PLoS One. 2016 May 19;11(5):e0155589. doi: 10.1371/journal.pone.0155589 (PMC4873262; doi:10.1371/journal.pone.0155589)
Supplement: S1 Table — Shinjoh in vitro experimental viral load data. (PDF) [file pone.0155589.s002.pdf]

Shinjoh in vitro experimental viral load data.

	Single infection		Simultaneous infection		Delayed Coinfection		
**Time** (d)	**IAV** (PFU/mL)	**RSV** ($TCID_{50}$/mL)	**IAV** (PFU/mL)	**RSV** ($TCID_{50}$/mL)	Delay (h)	**IAV** (PFU/mL)	**RSV** ($TCID_{50}$/mL)
0	0.0	0.0	0.018	0.00	0	7.3	3.3
1	6.41	2.49	6.29	2.44	4.0	7.0	3.7
2	7.91	4.19	8.12	2.50	8.0	6.0	4.5
3	8.21	4.24	7.79	1.70	12.0	5.7	5.0
4	7.93	4.50	7.60	0.99			
7	7.50	4.28	7.24	0.96			

All viral titer measurements are presented as $\log_{10}$ values.
